# Supplementary material for: SAMHD1 Attenuates Acute Inflammation by Maintaining Mitochondrial Function in Macrophages via Interaction with VDAC1
Source: Int J Mol Sci. 2023 Apr 26;24(9):7888. doi: 10.3390/ijms24097888 (PMC10177872; doi:10.3390/ijms24097888)
Supplement: Supplementary file 1 [file ijms-24-07888-s001.zip › Supplementary_Material.pdf]

Supplementary Material

SAMHD1 attenuates acute inflammation by maintaining mitochondria function in macrophages via interaction with VDAC1

Bowen Xu [1,†], Han Hu [1,2,†], Qianyi Sui [1,†], Xiangjia Hu [1], Xuchang Zhou [1,3], Xiaoming Deng [2,*], Cheng Qian [1,*], Nan Li [1,*]

†These authors contributed equally to this work.

***Correspondence:** Prof. Nan Li (N.L.) [1,*], nanli@immunol.org; Prof. Xiaoming Deng (X.D.) [2,*], dengphd@smmu.edu.cn; A/Prof. Cheng Qian (C.Q.) [1,*], crystalqiancheng@163.com

1 Supplementary Data

Results of co-immunoprecipitation coupled to mass spectrometry (IP-MS) has been presented in two Excel files, Data Sheet 1 represents IP conducted with Control IgG antibody. Data Sheet 2 represents IP conducted with Samhd1 antibody.

2 Supplementary Tables

Primer sequences of qPCR	
**mIL-6 Forward**	CTGCAAGAGACTTCCATCCAG
**mIL-6 Reverse**	AGTGGTATAGACAGGTCTGTTGG
**mTNFα Forward**	CTGAACTTCGGGGTGATCGG
**mTNFα Reverse**	GGCTTGTCACTCGAATTTTGAGA
**mIFNβ Forward**	CAGCTCCAAGAAAGGACGAAC
**mIFNβ Reverse**	GGCAGTGTAACTCTTCTGCAT
**mβ-actin Forward**	GGCTGTATTCCCCTCCATCG
**mβ-actin Reverse**	CCAGTTGGTAACAATGCCATGT

Supplementary Table S1. Primer sequences of qPCR

Antibody information		
**Samhd1 mouse mAb for WB and IF**	ab128107	Abcam, UK
**Samhd1 rabbit pAb for co-IP**	12586-1-AP	Proteintech, China
**Vdac1 rabbit pAb for IF**	55259-1-AP	Proteintech, China
**Vdac1 rabbit mAb for WB**	A19707	Abclonal, China
**Vdac2 rabbit mAb for WB**	A21260	Abclonal, China
**β-Tubulin Rabbit mAb**	A12289	Abclonal, China
**Phospho-IKKα/β (Ser176/180) rabbit mAb**	2697T	CST, USA
**IKKα rabbit mAb**	a19694	Abclonal, China
**Phospho-IκBα (Ser32) (14D4) rabbit mAb**	2859S	CST, USA
**IκBα (44D4) rabbit mAb**	4812S	CST, USA
**Phospho-NF-κB p65 (Ser536) (93H1) Rabbit mAb**	3033S	CST, USA
**NF-κB p65 (D14E12) XP® Rabbit mAb**	8242S	CST, USA
**Phospho-TBK1 (Ser172) (D52C2) XP® Rabbit mAb**	5483S	CST, USA
**TBK1/NAK (E8I3G) Rabbit mAb**	38066S	CST, USA

**Phospho-Erk1/2 MAPK (Thr202/Tyr204) mouse mAb**	9106S	CST, USA
**p44/42 MAPK (Erk1/2) Antibody**	9102S	CST, USA
**Phospho-p38 MAPK (Thr180/Tyr182) Antibody**	9211S	CST, USA
**p38 MAPK Antibody**	9212S	CST, USA
**Phospho-JNK (Thr183/Tyr185) (81E11) rabbit mAb**	4668S	CST, USA
**JNK2 (56G8) Rabbit mAb**	9258S	CST, USA
**AF647 anti-mouse F4/80 Antibody for FC**	123122	Biolegend, USA
**BV421 anti-mouse/human CD11b Antibody for FC**	101235	Biolegend, USA
**FITC anti-mouse CD80 Antibody for FC**	104706	Biolegend, USA
**AF700 anti-mouse CD86 Antibody for FC**	105122	Biolegend, USA
**AF700 anti-mouse CD45.2 Antibody for FC**	56-0454-81	Invitrogen, USA
**Rat Anti-Mouse CD16/CD32 Antibody**	553141	BD, USA
**PE anti-mouse CD206 (MMR) Antibody for FC**	141706	Biolegend, USA

Supplementary Table S2. Antibody information

3 Gating stragety, single controls and fluorescence-minus-one (FMO) controls of flow cytometry (FCM)

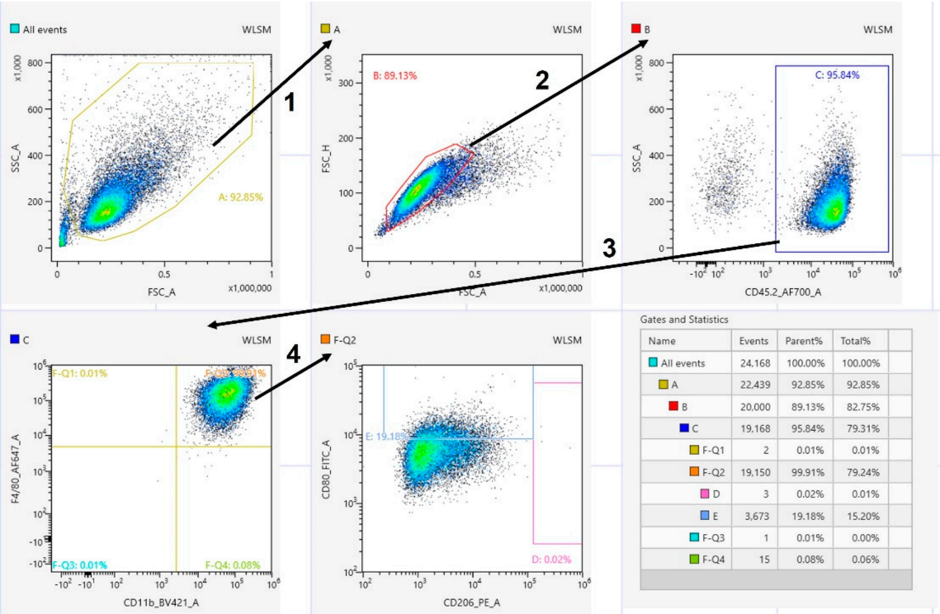

Supplementary Figure S2. Gating strategy of M1/M2 polarization FCM.

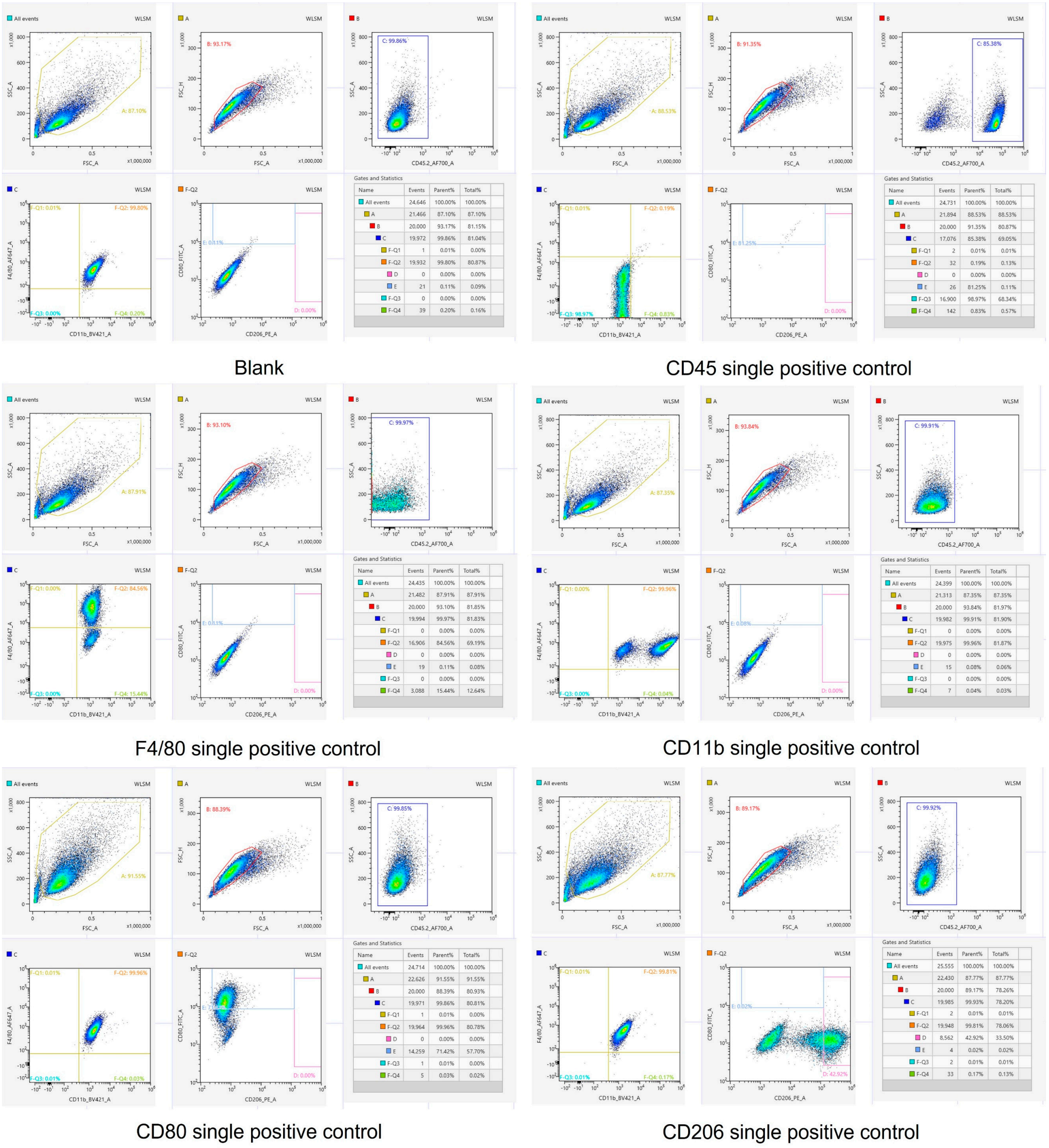

Supplementary Figure S2. Single positive controls of M1/M2 polarization FCM.

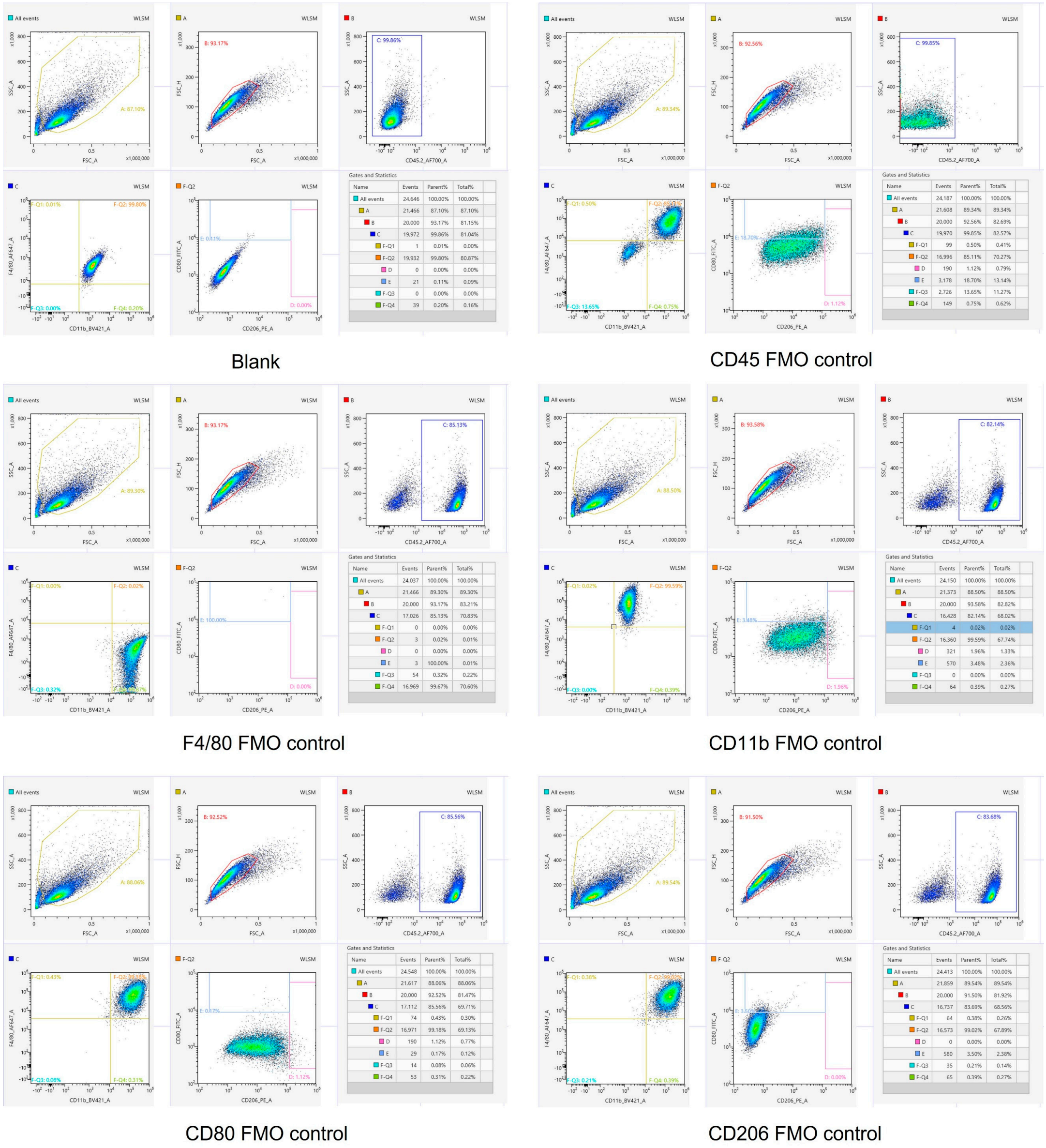

Supplementary Figure S3. FMO controls of M1/M2 polarization FCM.

4 Expression efficiency of the mutant plasmids

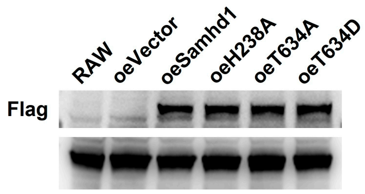

Supplementary Figure S4. Re-expression efficiency of SAMHD1 and its mutants detected by Flag tag.
